# Transient defects of mitotic spindle geometry and chromosome segregation errors

**DOI:** 10.1186/1747-1028-7-19

**Published:** 2012-08-11

**Authors:** William T Silkworth, Daniela Cimini

**Affiliations:** 1 Department of Biological Sciences, Virginia Tech, 1981 Kraft Dr, Blacksburg, VA, 24061, USA

**Keywords:** Mitosis, Mitotic spindle, Kinetochore, Centrosome, Chromosomal instability, Cancer, Aneuploidy

## Abstract

Assembly of a bipolar mitotic spindle is essential to ensure accurate chromosome segregation and prevent aneuploidy, and severe mitotic spindle defects are typically associated with cell death. Recent studies have shown that mitotic spindles with initial geometric defects can undergo specific rearrangements so the cell can complete mitosis with a bipolar spindle and undergo bipolar chromosome segregation, thus preventing the risk of cell death associated with abnormal spindle structure. Although this may appear as an advantageous strategy, transient defects in spindle geometry may be even more threatening to a cell population or organism than permanent spindle defects. Indeed, transient spindle geometry defects cause high rates of chromosome mis-segregation and aneuploidy. In this review, we summarize our current knowledge on two specific types of transient spindle geometry defects (transient multipolarity and incomplete spindle pole separation) and describe how these mechanisms cause chromosome mis-segregation and aneuploidy. Finally, we discuss how these transient spindle defects may specifically contribute to the chromosomal instability observed in cancer cells.

## 

Mitosis, the process by which a single eukaryotic cell partitions its genetic material, has fascinated scientists for over a century, and already in the late 1800s Walther Flemming described the processes of chromosome segregation and cell division with exquisite detail [1,2]. Four main structures, consisting of centrosomes, a microtubule-based mitotic spindle, kinetochores, and chromosomes [3,4], cooperate to form the mitotic apparatus (Figure 1A) in vertebrate somatic cells. The centrosomes are specialized organelles, each consisting of a pair of centrioles and pericentriolar material, and they play a key role in mitotic spindle assembly by serving as the primary sites of microtubule nucleation [5,6]. Molecular motors act to move the replicated centrosomes to diametrically opposing positions around the nucleus [7-9] (Figure 1A, Prophase), thus ensuring assembly of a fusiform and symmetric microtubule-based mitotic spindle once the nuclear envelope breaks down. At the same time, within the nucleus, the chromosomes undergo significant condensation (Figure 1A, Prophase) while kinetochores assemble on the centromeric region of each sister chromatid of the replicated chromosomes (reviewed in [10]). Upon nuclear envelope breakdown, which marks the beginning of prometaphase, the kinetochores become available for capture by dynamically searching microtubules (Figure 1A, Prometaphase). The kinetochore is a large protein complex that constitutes the attachment site for microtubules of the mitotic spindle on each chromatid [11]. In addition to acting as attachment sites for microtubules, kinetochores are also part of a signaling pathway, termed the spindle assembly checkpoint (SAC), that facilitates the coordinated and accurate segregation of chromosomes by preventing anaphase onset until all kinetochores are bound to microtubules (reviewed in [12]). As mitosis progresses, chromosomes establish attachments with microtubules and undergo poleward and anti-poleward movements, which are generated by minus end and plus end directed motors located at the kinetochore as well as along the chromosome arms [13-19]. Eventually, all chromosomes align between the two centrosomes, at the equator of the mitotic spindle, forming the metaphase plate (Figure 1A, Metaphase). Upon chromosome alignment, the SAC becomes satisfied [20], and the sister chromosomes segregate and move towards opposite spindle poles (Figure 1, Anaphase). All of these events must occur in a highly coordinated manner for accurate chromosome segregation into the two daughter cells. If any aspect of this process goes awry, cells may end up with an incorrect number of chromosomes, a state referred to as aneuploidy, which is the leading cause of mis-carriage and birth defects in humans and is a hallmark of cancer (reviewed in [21,22]). Thus, fidelity of the mitotic process is important for development and growth, as well as for homeostasis, repair, and renewal of adult tissues. In this review, we will focus on how defects in mitotic spindle geometry affect the fidelity of mitosis and how transient spindle geometry defects contribute to chromosomal instability in cancer cells.

**Figure 1:**
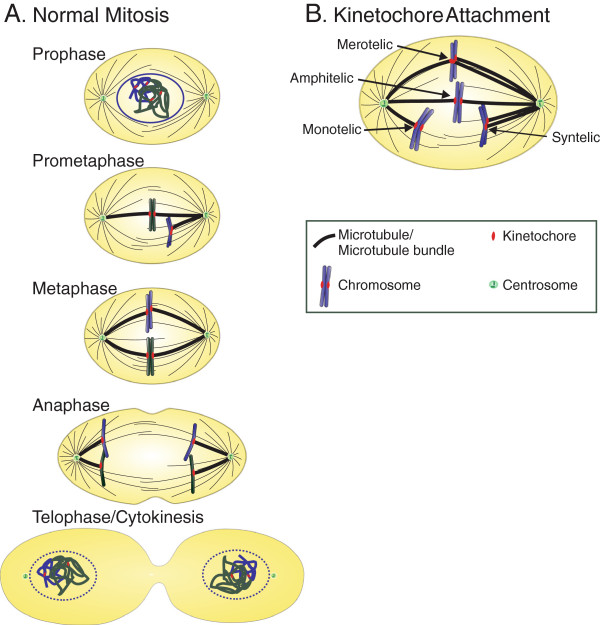
**Diagrammatic representation of mitosis, the mitotic apparatus, and different types of kinetochore attachments. A**. During the first stage of mitosis (prophase), the replicated chromosomes, still enclosed by the nuclear envelope, undergo condensation, while the replicated centrosomes move to diametrically opposing positions around the nucleus. Nuclear envelope breakdown marks the beginning of prometaphase, when kinetochores establish attachment with microtubules of the mitotic spindle. At the end of prometaphase, the chromosomes become aligned at the spindle equator forming the metaphase plate, and the cell is said to be in metaphase. The sister chromatids separate from each other and move to opposite poles of the mitotic spindle in anaphase. During the last stage of mitosis, telophase, the nuclear envelope begins to reassemble around the recently segregated chromosomes. Mitotic chromosome segregation is followed by cytokinesis, in which an actin-myosin contractile ring cleaves the cytoplasm to generate two individual daughter cells. **B**. Kinetochores and chromosomes can establish different types of attachments with microtubules during the early stages of mitosis. Monotelic attachment occurs when one sister kinetochore is attached to microtubules and the other sister is unattached. This is a typical first step in establishment of attachment during prometaphase. When the unattached sister kinetochore binds microtubules from the opposite spindle pole, the chromosome establishes amphitelic attachment. Amphitelic attachment is the only type of attachment that ensures correct chromosome segregation. Due to the stochastic nature of kinetochore-microtubule interactions, chromosomes can occasionally establish erroneous attachments. These include syntelic attachment, in which the two sister kinetochores bind microtubules from the same spindle pole, and merotelic attachment, in which a single kinetochore binds microtubules from both spindle poles instead of just one. Persistence of merotelic attachment into anaphase causes a chromosome segregation defect in the form of a lagging chromosome (see text and Figure 2 for details).

## Abnormal mitotic spindle geometry: permanent vs. transient

The bipolar geometry of the mitotic spindle is essential for accurate chromosome segregation, and already a century ago Theodor Boveri postulated that supernumerary centrosomes could lead to the production of aneuploid cells [23,24]. Observations of mitosis in both transformed and non-transformed cells reveal that multipolar mitotic spindles do occasionally form [25-31]. Typically, chromosomes within multipolar spindles form metaphase plates that display branched, Y-, V-, or T-shaped configurations as a consequence of chromosome alignment between multiple spindle poles [25,26]. Although chromosomes can align between supernumerary spindle poles, multipolar cell division has been shown to cause cell death in the progeny [32], most likely due to the fact that it causes massive chromosome mis-segregation, thus producing daughter cells that have far fewer chromosomes than is needed for survival. Moreover, anaphase lagging chromosomes (chromosomes that lag behind while all the other chromosomes segregate to the spindle poles during anaphase) are also frequently observed during multipolar cell division [25,27], thus adding to the burden of chromosome mis-segregation in multipolar cell division. Given the risk, it is not surprising that multipolar spindle assembly is a rare event in non-transformed cells growing under optimal conditions. Conversely, multipolar spindle assembly is very common in cancer cells [28-30,33-38], yet cancer cells avoid multipolar cell division and subsequent cell death by exploiting a number of mechanisms that allow centrosome clustering prior to anaphase onset [32,39-41]. Microtubule-associated proteins (e.g., NuMA, TPX2, ch-TOG, and ARL2), motor proteins (e.g., dynein and HSET), central spindle components, CLASPs, components of the Augmin complex, anillin, proteins involved in sister chromatid cohesion, kinetochore components, and chromosomal passenger proteins have all been implicated in the clustering of centrosomes prior to chromosome segregation [40-45]. The molecular and structural requirements that cause some cells to undergo multipolar anaphase and others to cluster their centrosomes are not yet know. However, variations in expression levels of minus end directed motors that oppose motors which serve to separate the centrosomes are likely involved. Alternatively, the orientation of the centrosomes as well as their distance from one another may serve to facilitate supernumerary centrosome clustering. These possibilities have yet to be explored, but studies investigating these issues would undoubtedly lead to a more comprehensive understanding of the specific mechanism(s) responsible for centrosome clustering. Regardless of the specific mechanisms, however, it is clear that cancer cells employ strategies to avoid permanent multipolarity, thus experiencing this mitotic spindle defect only transiently.

Another type of abnormal spindle geometry is observed in prometaphase cells with unseparated or incompletely separated spindle poles. In these cells, the centrosomes fail to migrate to opposing positions around the nucleus (a process driven by dynein and kinesin-5; reviewed in [46]) before the cell enters prometaphase (i.e., before nuclear envelope breakdown, NEB). If the centrosomes persisted in such unseparated/partially separated configuration, the cell would arrest in mitosis with a monopolar spindle, which would then lead to either mitotic slippage or cell death [47-49]. However, all cell types studied to date appear capable of completing centrosome separation after NEB thanks to a number of mechanisms, including Eg5 motor activity [46,50], myosin activity at the cell cortex [50,51], and kinetochore/kinetochore-microtubule-generated forces [52,53]. Once again, cells seem to have developed ways to avoid a permanent spindle defect and to limit monopolarity to a transient stage. The phenomenon of incomplete centrosome separation at NEB has been observed in a variety of cell types [52,54-56] and, while it was initially described in the mid- 1970s [55,56], only sporadic reports described this centrosome behavior over a period of several decades [51,54]. Recently, this phenomenon has received renewed attention [53,57] in part due to its correlation with increased rates of chromosome mis-segregation [57,58]. Why in some cells the centrosomes can reach diametrically opposing positions around the nucleus prior to NEB, whereas in other cells they cannot, is not clear. However, it has been proposed that the cause may be a lack of coordination between the timing of NEB and centrosome separation [55-57]. In any case, these cells achieve complete centrosome separation after NEB with no noticeable defects in subsequent mitotic spindle assembly or in the timing of mitosis as defined by the time between NEB and anaphase onset [52,55,57-59]. Nevertheless, cells that complete centrosome separation after NEB will experience monopolarity or near-monopolarity (spindle geometry defect) over a time window in early prometaphase, when initial kinetochore-microtubule attachments are being established.

## Transient spindle geometry defects, kinetochore mis-attachments, and aneuploidy

**Figure 2:**
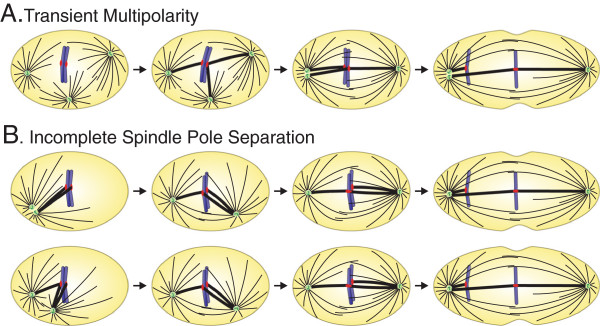
**Transient defects of mitotic spindle geometry and chromosome mis-segregation. A**. Transient multipolarity mechanism, in which initial assembly of a multipolar spindle (first panel) favors the formation of merotelic kinetochore attachment (second panel). Subsequently, centrosome clustering/coalescence leads to mitotic spindle bipolarization (third panel). However, merotelic kinetochore attachment can persist into anaphase and produce a chromosome segregation defect in the form of an anaphase lagging chromosome (fourth panel). **B**. Incomplete spindle pole separation at NEB results in a transient spindle geometry defect that promotes formation of kinetochore mis-attachments. If the centrosomes are very close to one another (top row), chromosomes are extremely likely to form syntelic attachments (first panel), which can be converted into merotelic attachments upon spindle bipolarization (second panel). If the centrosomes are not completely separated, but more than a few microns apart (bottom row), merotelic attachments can form directly without transitioning through a syntelic intermediate (first and second panel). In both cases, merotelic attachments can persist through mitosis (third panels) and induce chromosome mis-segregation in the form of anaphase lagging chromosomes (fourth panels). Adapted from [57].

Both examples of transient spindle geometry defects (transient multipolarity and incomplete/delayed spindle pole separation) described above cause high rates of kinetochore mis-attachment (Figure 1B) formation during prometaphase and lagging chromosomes [32,39,57,58] in bipolar anaphase cells (Figure 2).

Studies in various types of cancer cells showed that multipolar prometaphase cells display higher numbers of merotelic kinetochore attachments (one kinetochore bound to microtubules from two spindle poles instead of just one) compared to bipolar prometaphases ([32,39]; Figure 2A). Moreover, both experimental and computational analysis showed that the number of merotelic kinetochores increases with increasing numbers of spindle poles [60]. To explain this correlation, it was proposed that the reduced distance between each pair of spindle poles would increase the likelihood of each kinetochore to be reached by and bind to microtubules from two spindle poles instead of just one [32,39].

Experimental and computational studies were also employed to investigate the process of establishment of kinetochore attachment in cells with incomplete centrosome separation at NEB. These studies showed that when the two spindle poles are very close to each other upon NEB, chromosomes are extremely likely to establish syntelic attachments (both sister kinetochores bound to microtubules from the same spindle pole) ([57]; Figure 2B, top). As the spindle poles separate, such syntelic attachments can be partially corrected and converted into merotelic attachments ([57]; Figure 2B, top). When, upon NEB, the spindle poles are farther apart, but not diametrically opposed, kinetochores can form merotelic attachments without transitioning through a syntelic intermediate ([57]; Figure 2B, bottom). What is important to emphasize here is that in both cases of spindle geometry defects (multipolarity and near-monopolarity), large numbers of merotelic kinetochores are formed before spindle bi-polarization (Figure 2). Because the SAC cannot detect merotelic kinetochore attachment [61-65], cells can progress through mitosis in the presence of large numbers of merotelic kinetochores. Although merotelic kinetochores can be corrected by an Aurora B-dependent mechanism [66,67], mitosis will not halt to allow for correction, and therefore high rates of merotelic kinetochore formation invariably result in high rates of anaphase lagging chromosomes (reviewed in [21]). Indeed, both transient multipolarity and incomplete spindle pole separation have been shown to result in high rates of anaphase lagging chromosomes ([32,39,58]; Figure 2). Thus, abnormal spindle geometry, albeit transient, can have detrimental effects on the fidelity of chromosome segregation, and represents a potential source of aneuploidy. Interestingly, over 70% of cancer cells from various sites are aneuploid [21,68], and many of them also display high rates of chromosome mis-segregation, a phenotype that leads to continuous changes in chromosome number, or *c*hromosomal *in*stability (CIN) [22,69-71]. Recent studies have shown that merotelically attached anaphase lagging chromosomes represent the most common chromosome segregation defect in CIN cancer cells [32,39,72]. One cause of such high rates of anaphase lagging chromosomes appears to be the inefficiency of the correction mechanisms for kinetochore mis-attachments in cancer cells [73]. However, the high rates at which kinetochore mis-attachments (particularly merotelic) form are perhaps the major cause of chromosome mis-segregation in cancer cells, and the transient spindle geometry defects described above represent the most likely mechanisms of kinetochore mis-attachment formation in cancer cells ([32,39]; Silkworth, Nardi, and Cimini, unpublished; see below for further discussion).

## Transient spindle geometry defects in development, adult tissues, and cancer

The transient spindle geometry defects described here and their effects on the fidelity of chromosome segregation have been mainly characterized in tissue culture cells, with transient multipolarity exclusively observed in CIN cancer cells to date. Indeed, the frequencies of multipolar spindles in non-transformed or non-CIN cancer cells are typically very low (Silkworth, Nardi, and Cimini, unpublished). Given the causal relation between transient multipolarity and chromosome mis-segregation and that transient multipolarity is very common in CIN cancer cells, this mechanism is widely recognized as a major player in CIN. Whether this mechanism is also acting in tumors *in situ* has not been investigated. However, multipolar spindles have been observed in tumor tissues [74-76] and short-term tumor cell cultures [77,78].

Although incomplete spindle pole separation at NEB has also been characterized mainly in tissue culture cells, the information available to date reveals that this mechanism is not exclusive to cancer cells, and has indeed been observed in several different types of tissue culture cells at frequencies of ~40-45% [51,52,55,57,59]. Moreover, studies aimed at investigating various aspects of cell division provide useful information on the occurrence and relevance of this mechanism in contexts other than tissue culture. For example, in both the one- and two-cell stage of the *Caenorhabditis elegans* embryo, the centrosomes achieve opposing positions around the nucleus before the nuclear envelope breaks down [79]. Similarly, in the syncytial *Drosophila* embryo, the centrosomes always achieve diametric arrangement around prophase nuclei [80], and the nuclear envelope does not break down until the centrosomes have completed their movement around the nucleus [81]. This ability of cells in developing embryos to completely separate their centrosomes before NEB has also been observed in *Drosophila melanogaster* neurogenesis. Indeed, in both epidermoblasts and neuroblasts the centrosomes are completely separated before the onset of prometaphase [82]. Given the risk of chromosome mis-segregation associated with incomplete centrosome separation at NEB, it is not surprising that this defect is not observed during early development, as it would potentially lead to mosaic aneuploidy and possibly embryonic death.

The incidence of incomplete centrosome separation at NEB in normal adult tissues has not been investigated to date. However, a recent study showed that non-cancer RPE1 cells, which are known to maintain a stable karyotype with negligible rates of aneuploidy [72], always succeed to separate their centrosomes before NEB [83]. This observation suggests that centrosome separation in normal proliferating cells, like in developing embryo cells, may be better timed with NEB compared to cancer cells or certain stabilized tissue culture cells.

The open question, then, is whether incomplete centrosome separation at NEB may play a role in cancer cell CIN, and if so, to what extent. As discussed above, transient multipolarity is recognized as a major cause of chromosome mis-segregation in cancer cells [32,39,84]. However, some CIN cancer cell types cannot cluster the centrosomes of multipolar spindles very efficiently (Silkworth, Nardi, and Cimini, unpublished). Yet, these cells exhibit high rates of chromosome mis-segregation in the form of lagging chromosomes in bipolar anaphase cells (Silkworth, Nardi, and Cimini, unpublished), raising the possibility that incomplete spindle pole separation at NEB may play a role in promoting formation of kinetochore mis-attachments in these cells. Analysis of centrosome separation in early prometaphase shows that incomplete spindle pole separation at NEB can be observed in as many as 70% of the cells in those cancer cell lines that display inefficient centrosome clustering (Silkworth, Nardi, and Cimini, unpublished). These observations indicate that incomplete centrosome separation at NEB may play an important role in promoting CIN in certain cancer types.

## Conclusions

Accurate partitioning of chromosomes to the daughter cells during mitosis is of utmost importance to ensure development and growth of all eukaryotic organisms. Defects of the mitotic spindle have a dramatic impact on chromosome segregation. However, the effect on the cell population can be even more dramatic if the spindle defects are only transient. Indeed, whereas permanent spindle defects typically lead to cell death in the progeny, transient spindle defects increase the rates of chromosome mis-segregation, but not to a level that would affect cell viability, thus ultimately being more threatening to the overall cell population and/or the organism. Here, we have discussed two types of transient mitotic spindle defects that are associated with increased rates of kinetochore mis-attachment formation and chromosome mis-segregation. One of them, transient multipolarity, is currently recognized as a common mechanism of CIN in cancer cells [32,39,84]. Conversely, the incomplete centrosome separation (at NEB) mechanism appears to occur at moderate levels in many different types of cancer cells, but it also occurs at very high frequencies in cells from certain types of cancers. This is a very interesting observation that needs further investigation. For example, it would be interesting to study whether the incidence of this mechanism relates to cancer progression or whether it is typical of cancer cells from specific sites. Moreover, the fact that incomplete centrosome separation at NEB is also observed in several non-transformed cell types suggests the possibility that this mechanism may occur in normal proliferating somatic cells and may, through its ability to promote chromosome mis-segregation, play a role in tumor initiation. This possibility undoubtedly deserves consideration in the near future. Future studies should also be focused on the causes of incomplete centrosome separation at NEB. It is plausible to imagine that mis-regulation of key motor proteins may be at the basis of this defect. An alternative hypothesis is that the ability of the centrosomes to separate in a timely fashion is dictated by signals from and/or physical interactions with trans-membrane elements. For example, it is widely acknowledged that dividing cells within polarized epithelia rely on such mechanisms to orient the mitotic spindle [85-87]. Similar mechanisms may dictate not only the exact positioning of the centrosomes, but also the timing of centrosome separation. One last possibility is that centrosome separation *per se* is not impaired in cells with incomplete centrosome separation at NEB, but the exact timing is inaccurate, so that centrosome separation and NEB are no longer coordinated. It is possible that such coordination is finely regulated at the early stages of development [50,80,81,88], but is lost in the adult. In fact, optimal timing between centrosome separation, NEB, and chromosome segregation is very important during the early stages of development to ensure the chromosomal stability necessary to the development of a healthy adult organism. In conclusion, incomplete centrosome separation at NEB is a newly characterized mechanism of CIN for which we still have numerous open questions, and as such it will likely become the focus of many studies in the near future.

## Abbreviations

SAC: Spindle Assembly Checkpoint; NEB: Nuclear Envelope Breakdown; CIN: Chromosomal INstability.

## Competing interests

The authors declare that they have no competing interests.

## Authors’ contributions

WTS and DC conceived the ideas and wrote the paper. Both authors read and approved the final manuscript.

## Authors' information

WTS was a PhD student in the Department of Biological Sciences at Virginia Tech, and completed his dissertation work in July 2012 under the guidance of Dr. Daniela Cimini.

DC is an Associate Professor in the Department of Biological Sciences at Virginia Tech.
